# Enhancement of Pea–Oat Composite Protein Gel Properties Through Ultrasound Treatment Affects Structural and Functional Characteristics

**DOI:** 10.3390/foods14213751

**Published:** 2025-10-31

**Authors:** Sai Wang, Mengxiao Li, Guimei Dong, Ruiling Shen, Jilin Dong, Yunlong Li

**Affiliations:** 1 School of Food and Biological Engineering, Zhengzhou University of Light Industry, Zhengzhou 450002, China; wangsaizzuli@163.com (S.W.); limengxiao2022@163.com (M.L.); shenrl1967@163.com (R.S.); 2 School of Food and Health Engineering, Zhengzhou University of Technology, Zhengzhou 450044, China; d18503835079@163.com; 3 China Institute of Functional Food of Shanxi, Shanxi Agricultural University, Taiyuan 030031, China

**Keywords:** POPG, ultrasonic treatment, rheological, thermal stability, microstructure

## Abstract

With increasing attention to health, plant protein products have gained significant market potential due to their growing consumer demand. This study researches the influence of ultrasonic treatment on the structure and function of pea–oat composite protein gel (POPG) to enhance its elasticity and thermal stability. The ultrasonic treatment parameters were regulated to power (200–600 W for 30 min) and ultrasonic time (20–40 min at 400 W) during the preparation of POPG, and the properties and structure, including gel strength, rheological analysis, water-holding capacity (WHC), thermal characteristics, fluorescence performance, and microstructure, were further evaluated. The results showed that the POPG samples exhibited optimal values in WHC, gel strength, surface hydrophobicity, free sulfhydryl amount, and endogenous fluorescence at 400 W ultrasonic for 30 min compared with the untreated POPG. Rheological analysis indicated that POPG displayed the highest storage modulus and improved viscoelasticity. Ultrasonication resulted in an augmentation in β-sheet content, hence creating a more compact network structure. DSC and TGA revealed improved thermal stability, while SEM and CLSM exhibited a homogeneous and firm gel structure of POPG. This research offers the theory that ultrasonic technology can improve the performance of plant-based composite gels.

## 1. Introduction

The growing demand for sustainable food production has accelerated the substitution of animal-derived ingredients with plant-based alternatives in food processing. Previous life-cycle assessments indicate that replacing 50% of animal-source proteins with plant-based analogues can decrease diet-related greenhouse gas emissions by 35% and agricultural land use by 56% [1]. Lately, plant proteins are receiving heightened focus as a viable alternative to animal proteins within the food processing sector and consumer market, owing to their abundant resources, cost-effectiveness, and high nutritional value [2]. Among numerous plant protein sources, peas and oats have emerged as key raw materials for development in the food sector, given their specific nutritional compositions, functional characteristics, and excellent processing adaptability. Pea protein, with a high protein content of 21–28%, has a relatively balanced amino acid composition but a relatively low methionine content [3]. However, amino acid complementation can be achieved by blending with oat protein, thereby enhancing the overall nutritional value. Moreover, pea protein is gluten-free and cost-effective, exhibiting excellent functional properties in food processing, including emulsification capacity, gelation capacity, and water-holding capacity, which makes it an ideal choice for gel-based products [4,5].

Oats have a protein content of 12–20%, and their essential amino acid composition is more balanced compared to other grains [6]. This protein possesses high digestibility and biological value, but its limited gelation capacity restricts its application in products [7]. Research indicates that the connections among several proteins may enhance the stability of gel networks during the gelation process while also improving the elasticity and toughness of gels and their structural compactness [8,9]. Therefore, compared to single-protein gels, composite protein gels exhibit superior gel texture and functional properties. Our research findings revealed that the pea–oat composite protein gel (POPG), prepared by mixing pea and oat proteins at a certain ratio, followed by the addition of transglutaminase (TG) and chitosan, exhibited satisfactory hardness and water-holding capacity (WHC). However, it still suffers from several drawbacks, including insufficient elasticity and inadequate thermal stability, which impede its wider use in food processing.

Ultrasound, being a non-thermal mechanical action method, has the characteristics of safety, high efficiency, and sustainable development and shows great potential in food applications [10]. Ultrasonic treatment induces conformational reorganization and aggregation state transition of protein molecules through cavitation effects and mechanical shear forces, diminishes the size of protein clusters, and consequently induces interactions and structural alterations within the protein, thereby effectively regulating the structure and function of gel systems. These alterations resulted in the exposure of reactive groups, including the development of sulfhydryl groups and hydrophobic residues, such as disulfide and dityrosine linkages. This alteration is strongly related to the increase of protein functional qualities, including thermal properties, gel strength, and water retention [11,12]. For instance, Gao, X. et al. [13] reported that ultrasound effectively improved the properties and microstructure of low-salt surimi gel. Xin, Y. et al. [14] reported that the ultrasonic pretreatment can markedly improve the gel strength of soy protein modified with emulsified coagulant and facilitates the development of a stable and homogeneous network structure. Liu, B. et al. [15] demonstrated that high-frequency ultrasonics effectively increased the gel strength, viscoelasticity, and WHC of myofibrillar protein.

The integration of plant proteins into processed foods can enhance high-quality protein content and substitute fat in certain products due to their superior gelling and viscoelastic capabilities, hence facilitating the development of healthier formulations. The current study indicates that research on soybean protein within the legume-cereal composite protein system has advanced; nevertheless, its application in food processing remains constrained due to anti-nutritional effects and the pronounced bean flavor of soybean. Consequently, we endeavored to utilize pea protein as the foundational legume protein, in conjunction with oat protein, to develop a novel plant protein gel and investigate its potential applications in food processing.

Despite our previous optimization of POPG system via enzyme hydrolysis followed by chitosan modification [16], the attributes of POPG are still unsatisfactory. This study is the first to incorporate ultrasound treatment, thereby filling the knowledge gap regarding ultrasound-induced structural and functional regulation of this system, hence enhancing the properties of POPG, aiming to evaluate the feasibility of ultrasonic treatment in the processing of plant protein gel goods.

## 2. Materials and Methods

### 2.1. Materials

The oat bran was sourced from Shanxi Huaqishun Food Technology Co., Ltd. (Linyi, China). Pea protein (purity of 92.1%) was purchased from Yawing Ecological Food Industry Co., Ltd. (Yucheng, China). Transglutaminase (TG, 10,000 U/g) was provided by Dongsheng Biotechnology Co., Ltd. (Taixing, China). Chitosan (DDA is 96.1%, Mw ≈ 200 kDa) was procured from Wananga Chemical Technology Co., Ltd. (Zhengzhou, China). All chemical reagents were acquired from Sinopharm Chemical Reagent Co., Ltd. (Shanghai, China).

### 2.2. Preparation of Oat Protein

Oat protein was made in the laboratory. Oat bran and deionized water were combined in a 1:10 (g:mL) ratio, with the addition of 1.5% β-glucanase. The pH was modified to 6.5 using a NaOH solution (1.0 mol/L), and the enzymatic hydrolysis was conducted at 50 °C for 30 min. The pH was modified to 9.6 using NaOH solution, followed by magnetic stirring for 2 h, centrifugation at 10,000 rpm at 4 °C for 10 min, and collection of the supernatant. The pH of the supernatant was adjusted to 4.4 using a 1.0 mol/L HCl solution, then centrifuged at 10,000 rpm at 4 °C for 10 min. The protein precipitate was thereafter collected and washed twice with water. The pH was modified to 7.0 using NaOH solution to redissolve the protein, after which the protein was freeze-dried. The yield was 16.14% based on the protein content in bran, and the purity of oat protein was 73.62%, as determined by the Kjeldahl method.

### 2.3. Preparation of POPG by Ultrasonic Treatment

The preparation process of POPG was as follows: Briefly, pea protein and oat protein were combined with deionized water at a ratio of 1:0.4 to prepare a 20% (*w*/*w*) aqueous solution. We used a SCIENTZ-IID ultrasonic cell disruptor (Ningbo Scientz Biotechnology Co., Ltd., Ningbo, China) for the ultrasonic treatment. Two sets of experiments were conducted: (1) the power was fixed at 400 W, and the time was varied (20, 25, 30, 35, and 40 min); (2) the ultrasonic time was fixed at 30 min, and the power was varied (200, 300, 400, 500, and 600 W). After ultrasonic treatment, TG (40 U/g) was blended into the protein solution and completely dissolved. The hybrid was then maintained at 45 °C for 1 h by a HH-S6 thermostatic water bath (Beijing Keweiyongxing Instrument Co., Ltd., Beijing, China). Next, chitosan powder was added directly into the dispersion to give a final concentration of 2% (*w*/*w*) relative to the total dry mass (pea + oat protein + chitosan), and the mixture was heated at 95 °C for 30 min, instantly cooled to room temperature using an ice-water bath, and then kept at 4 °C for 24 h to get the ultrasonicated POPG. The POPG without ultrasonic treatment served as the control.

### 2.4. Gel Strength Analysis

The gel strength test method was conducted according to Liu et al. [17] with slight modifications. The sample (diameter: 20 mm; height: 30 mm) was assessed using the XT Plus texture analyzer (Stable Micro Systems, Godalming, Surrey, UK). A TA10 probe was selected to move at a constant velocity of 1.0 mm/s, and the compression distance was 15 mm. The peak force measured during compression was designated as the strength (g) of the gel during the initial compression.

### 2.5. Water Holding Capacity (WHC) Analysis

The WHC of the sample was measured according to the method of Meng et al. [18]. The sample was sliced into small cubic pieces, and their original mass was accurately noted. The sample was then wrapped with filter paper and centrifuged at 8000 r/min for 10 min. The sample pieces were immediately removed and reweighed. The WHC was determined with the given formula:

(1) $$WHC(\%)=\frac{m_{2}}{m_{1}}\times 100\%$$

where m_1_ and m_2_ denote the mass (g) before and after centrifugation, respectively.

### 2.6. Rheological Analysis

#### 2.6.1. Frequency Sweep Test

The method of frequency sweep test was conducted according to Wu et al. [19] with modifications. The sample, cut into cylindrical shapes with a diameter of 1.5 mm and a height of 1 mm using a blade, was tested using a Discovery HR-1 rotational rheometer (TA Instruments Inc., New Castle, DE, USA) at 25 °C. A 40 mm parallel-plate fixture (model PP50) was employed via a 1 mm gap and 0.5% strain amplitude. The storage modulus (G′) and loss modulus (G″) were recorded at frequencies between 0.1 and 100 rad/s.

#### 2.6.2. Strain Sweep Test

The test method was modified from Taktak et al. [20]. The sample was tested with a 40 mm parallel-plate fixture (model PP50) at a fixed frequency of 1 Hz and temperature of 25 °C. A strain sweep ranging from 0.1% to 100% was conducted to measure the G′ and G″.

### 2.7. Thermal Stability Analysis

#### 2.7.1. Differential Scanning Calorimetry (DSC) Analysis

The method of the thermal characteristics test was modified from Sahraee et al. [21]. Briefly, 8 mg of freeze-dried sample within a sealed aluminum plate was heated from 30 to 120 °C at a rate of 5 °C/min under nitrogen by using a DSC 4000 heat-flow differential scanning calorimeter (PerkinElmer Instruments Co., Ltd., Shanghai, China). An empty aluminum plate served as the control. The T_o_ (onset temperature), T_p_ (peak temperature), and T_c_ (conclusion temperature) were noted, and ΔH (denaturation enthalpy value) was computed from integration of the peak area.

#### 2.7.2. Thermogravimetric (TGA) Analysis

The method for the thermal stability test was modified from Li et al. [22]. Briefly, by using a Mettler Toledo thermal gravimetric analyzer (Mettler Toledo International Inc., Greifensee, Switzerland), 8 mg of freeze-dried POPG was encapsulated in an aluminum plate and exposed to heating from 40 to 700 °C at an average speed of 10 °C/min under a nitrogen flow. The weight changes and the first derivative of the weight loss rate of the POPG samples were recorded during the heating process.

### 2.8. Free Sulfhydryl and Disulfide Bonds Analysis

The test method was determined according to Shen et al. [12] with slight modifications. Briefly, 15 mg of the sample was added to 5 mL of Tris-glycine buffer for analysis, while an additional 15 mg was dissolved in 5 mL of Tris-glycine-urea buffer for total sulfhydryl analysis. The mixture was centrifuged at 10,000 r/min for 10 min. Subsequently, 20 µL of Ellman’s reagent (4 mg/mL DTNB) was incorporated into 1 mL of each supernatant. The absorbance was measured at 412 nm. The calculation formulas for free sulfhydryl, total sulfhydryl, and disulfide bonds were as follows:

$$Free\;sulfhydryl\;(\mu mol/g)=\frac{73.53\;\times \;D\;\times \;A_{412}}{C}$$

$$Disulfide\;bonds\;(\mu mol/g)=\frac{Total\;sulfhydryl-free\;sulfhydryl}{2}$$

where A_412_ denotes the absorbance of the sample. The dilution factor (D) for the free sulfhydryl groups is 5.02, and the dilution factor for the total sulfhydryl groups is 10. C denotes the mass concentration of the sample (mg·mL^−1^).

### 2.9. Surface Hydrophobicity Analysis

The method of the surface hydrophobicity test was modified from Ma et al. [23]. In summary, 1.50 g of the sample was added to 15 mL of 10 mM phosphate buffer (pH 7.0) and centrifuged at 8000 r/min for 5 min. The upper aqueous phase was collected and diluted with the same buffer to obtain protein concentrations of 0.1, 0.2, 0.4, 0.6, and 0.8 mg/mL. An aliquot (2 mL) of each dilution was combined with 10 μL of 8 mM ANS solution and placed in the dark at room temperature for 15 min. The experiment was conducted with the excitation wavelength set at 390 nm and the emission wavelength set at 460 nm.

### 2.10. Intermolecular Forces Analysis

The test was according to the method of Zhu et al. [24] with slight modifications. The sample was added in four distinct solutions: 0.05 mol/L NaCl solution (S1), 0.6 mol/L NaCl solution (S2), 0.6 mol/L NaCl solution with 1.5 mol/L urea (S3), and 0.6 mol/L NaCl solution with 8 mol/L urea (S4). The mixture was centrifuged at 5000× *g* for 20 min, determining the protein concentration in the supernatant. The contributions of different intermolecular forces were calculated by measuring the absorbance at 595 nm with a Spectra Max M5 multi-mode microplate reader (Molecular Devices, San José, CA, USA) as follows: ionic bonds (S2-S1), hydrogen bonds (S3-S2), and hydrophobic interactions (S4-S3).

### 2.11. Endogenous Fluorescence Spectrum Analysis

The test was determined by using an SP-1920 fluorescence spectrophotometer (PerkinElmer Instruments Co., Ltd., Shanghai, China), and according to the method of Cao et al. [25]. The sample was prepared into a 0.5 mg/mL solution using 0.01 M phosphate buffer. Adjust the excitation wavelength to 280 nm and the emission wavelength to from 200 nm to 450 nm, the scanning speed was 1500 nm/min, and both the emission and excitation slits were 2.5 nm.

### 2.12. Fourier Transform Infrared Spectrophotometer (FTIR) Analysis

The FTIR scanning experiment was conducted on a PerkinElmer Spectrum Two FT-IR spectrometer (Waltham, MA, USA). The lyophilized POPG was combined with KBr at a mass ratio of 1:50, then crushed and compressed into translucent pellets. The ambient temperature was established at 25 °C, with a resolution of 4 cm^−1^ and a wave number precision of 0.01 cm^−1^, and the measurement was conducted within the wave number range of 4000–400 cm^−1^. The scanning dates were derived using OMNIC 9 (ThermoFisher Scientific, Waltham, MA, USA) software to rectify the baseline, with the sample data normalized to yield the absorbance and transmittance spectra. The FTIR spectra of the protein amide I band in the range of 1700–1600 cm^−1^ were deconvoluted and peak-separated utilizing PEAKFIT v4.12 (SeaSolve Software, San Jose, CA, USA), employing Gaussian solidification fitting of the nine-point Savitzky–Golay derivative function, alongside the protein secondary structure data derived from second-order derivative analysis and curve fitting.

### 2.13. Morphological Analysis

#### 2.13.1. Scanning Electron Microscope (SEM)

The POPG was freeze-dried, followed by gold coating for SEM analysis by using a Sigma 300 scanning electron microscope (Carl Zeiss AG, Oberkochen, Germany). Subsequently, the microstructure of the gel was analyzed at an operating voltage set at 3.0 kV and a magnification level of 20,000×.

#### 2.13.2. Confocal Laser Scanning Microscope (CLSM)

The imaging was observed using Carl Zeiss AG (Carl Zeiss AG, Germany). The sample with a thickness of 1 mm was immersed in 2.5% glutaraldehyde solution and then stored at 4 °C for 2 h. Subsequently, a 0.05% fluorescein isothiocyanate solution was used to stain the proteins, and the excitation wavelength was adjusted to 488 nm.

### 2.14. Statistical Analysis

All the assays were conducted three times, with outcomes represented as the mean ± standard deviation. SPSS 24.0 was used for one-way ANOVA with a significance level of *p* < 0.05. Data visualization was used for Origin 2024.

## 3. Results

### 3.1. Effects of Ultrasonic Treatment on the Gel Strength and WHC of POPG

**Figure 1:**
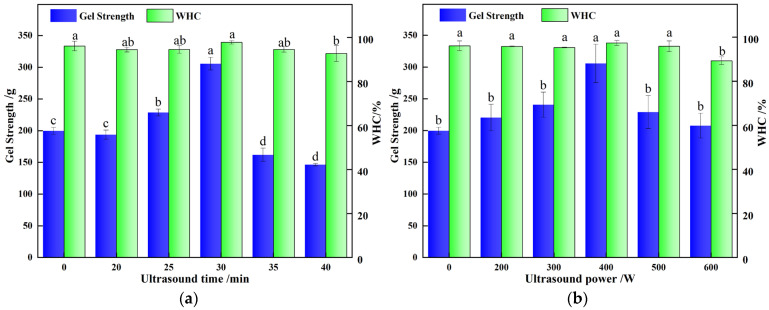
Effects of different ultrasonic treatments on gel strength and WHC of POPG: (**a**) Effects of different ultrasonic *time* on gel strength *and* WHC of POPG; (**b**) Effects of different ultrasonic *power* on gel strength and WHC of POPG. Different letters in the same column indicate significant differences (*p* < 0.05).

Gel strength and WHC are key indices of gel quality, reflecting network stability and water-retention ability, respectively. Figure 1a,b illustrate that the gel strength of ultrasonicated POPG samples initially increased followed by a decrease, compared with the control samples. In contrast, WHC showed no significant difference under varying ultrasonic conditions. This can be explained by the ultrasound treatment enhancing the stability and density of the gel network structure by tightening the network through modulated protein-protein interactions [10]. Conversely, excessive ultrasonic treatment (>400 W, >30 min) induced protein over-denaturation and disrupted the network, leading to a substantial deterioration in gel properties. Fu, Y. et al. [26] demonstrate a clear biphasic response in Hypomesus olidus surimi, where gel strength initially increases with ultrasonic power and duration up to an optimum point (200 W, 10 min), beyond which excessive treatment may lead to protein network degradation. Xin, Y. et al. [14] demonstrate that more sulfhydryl groups are exposed on the protein surface, which enhances the formation of disulfide bonds during tofu coagulation, thereby improving gel strength following ultrasound treatment. However, excessive ultrasonic power reduces the disulfide bonds in soy milk, converting them into sulfhydryl groups, which ultimately decreases the disulfide bond content in the tofu.

### 3.2. Effect of Ultrasonic Treatment on the Rheological Properties of POPG

**Figure 2:**
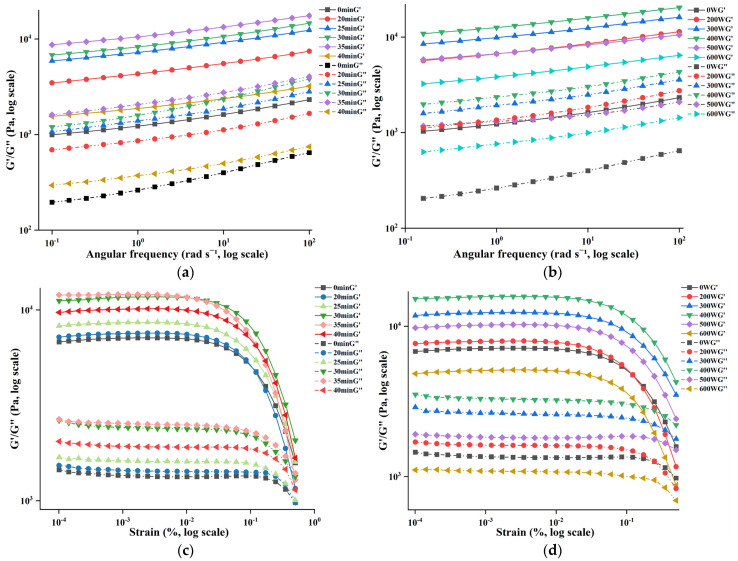
Effects of different ultrasonic treatments on the rheological properties of POPG: (**a**) Variation curves of G′ and G″ with angular frequency in different ultrasonic time; (**b**) Variation curves of G′ and G″ with angular frequency in different ultrasonic power; (**c**) Variation curves of G′ and G″ with strain in different ultrasonic time; (**d**) Variation curves of G′ and G″ with strain in different ultrasonic power.

Rheological measurements of G′ and G″ reveal the viscoelastic response and network integrity of the gel, offering quantitative theoretical support for the formulation, process optimization, preparation control, and shelf-life prediction of plant-based meat products, dairy products, and fat-based products in the food industry. As shown in Figure 2a,b, in the frequency range of 0.1–100 rad·s^−1^, the G′ of each sample always exceeded G″, which indicates pronounced elastic dominance within the linear viscoelastic region [27,28]. Each sample displayed an initial rise in G′ and G″, a reduction with increasing ultrasonic power, which exhibited pronounced frequency dependence, indicating typical viscoelastic behavior consistent with previous reports [29]. Moreover, when compared with the control samples, the G′ and G″ of ultrasonicated POPG samples were significantly elevated under varying ultrasonic conditions. This suggests that ultrasonic treatment can enhance the elastic and viscous characteristics of the gel network [30]. At 400 W ultrasonic power for 30 min, the G′ and G″ values were the highest, which was aligned with the conclusions mentioned above. This is due to the fact that ultrasonic treatment can decrease the gel granularity, increase dissolvability, and expose more active sites, thereby improving the gel strength of POPG [23].

Strain sweep quantifies gel deformation and failure characteristics, providing a theoretical basis for microstructural strength analysis and stability prediction of protein gels. As shown in Figure 2c,d, the strain curve represents the linear viscoelastic (LVE) condition, where, initially, G′ significantly exceeds G″, indicating that POPG possesses viscoelastic features. When the strain is above the LVE limit, the values of G′ and G″ gradually approach each other, attributable to the rupture of the internal bonds within the gel network under elevated strain [31]. Under varying ultrasonic conditions, the G′ of all samples exhibited an initial increase followed by a decrease, while G″ showed no significant change. When the treatment time was 30 min and the power was 400 W, both G′ and G″ in the strain sweep reached their maximum values. This phenomenon can be attributed to ultrasound-induced protein molecular rearrangement, which forms forming a densified cross-linked network, and thereby boosts the viscoelastic strength because of significantly enhancing G′. The ultrasonic treated POPG exhibited a more gradual modulus decline curve due to improved structural homogeneity [32]. Mozafarpour, R. et al. [33] reported that the emulsion gels treated with 300 W ultrasonic power were more resistant to fracture than the untreated.

### 3.3. Effect of Ultrasonic Treatment on Thermal Properties of POPG

DSC was conducted to evaluate the impact of ultrasonic processing on the thermal characteristics of POPG, including T_o_, T_p_, T_c_, and ΔH. T_p_ represents the peak temperature of protein denaturation, while ΔH represents the energy necessary for the denaturation process and can also evaluate the extent of protein unfolding [34]. As shown in Table 1, the protein denaturation peak temperature of ultrasonicated POPG samples was marked higher than that of the untreated with increasing ultrasonic power and treatment time, indicating that ultrasonic treatment increased the denaturation temperature of POPG. At an ultrasonic power of 400 W for 30 min, the T_p_ values reached their peaks at 106.21 °C and 110.99 °C, respectively. Correspondingly, ΔH increased from 1695.75 J/g to 1822.35 J/g and 1842.35 J/g. The ultrasonic treatment broke the intermolecular bonds of POPG due to cavitation and turbulence forces, exposing hidden hydrophobic groups on the surface. This increases hydrophobic interactions and forms a more stable structure, leading to an increase in ΔH [35,36]. Zhang et al. [37] reported that compared to the untreated samples, the peak temperature of protein denaturation increased from 128.2 °C to 131.9 °C after the soybean protein isolate and pectin emulsion gels were treated with ultrasound at 450 W.

**Figure 3:**
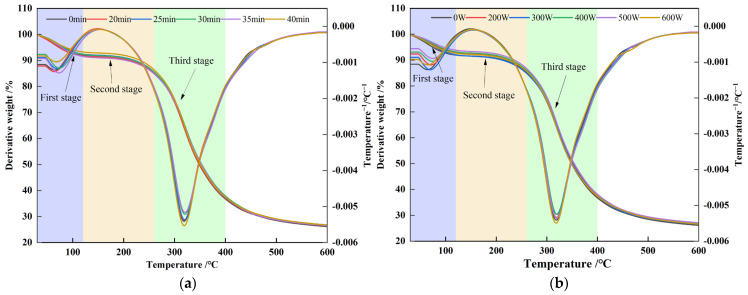
Effects of different ultrasonic treatments on the thermal stability of POPG: (**a**) Effects of different ultrasonic treatments time on the thermal stability of POPG; (**b**) Effects of different ultrasonic treatments power on the thermal stability of POPG. The different colors denote the three stages of the thermogravimetric evolution trend.

Thermal stability is a crucial determinant influencing the quality and shelf life of gel-type foods. Thermogravimetric analysis (TGA) of POPG was conducted within a temperature range from 40 °C to 700 °C with a heating rate of 10 °C min^−1^ to monitor the changes in mass during programmed heating. Figure 3a,b illustrate the weight loss curves of all samples, which indicated consistent trends through three distinct stages. In the first stage, from a temperature range of 40 °C to 120 °C, a minor weight loss occurs due to moisture evaporation [38]. In the second stage, which occurs between 120 and 260 °C, the secondary and tertiary structures of proteins are disrupted, during which weaker hydrogen bonds and some peptide bonds are cleaved, leading to more significant weight loss and the onset of structural disintegration [39]. The third stage, approximately from 260 to 400 °C, involves the breakdown of the protein backbone by cleaving stronger covalent bonds, releasing gases such as CO, CO_2_, NH_3_, and H_2_S [40], which results in a marked decrease in weight. Moreover, ultrasonicated POPG samples lost more mass than the untreated POPG under varying ultrasonic conditions. This decrease in weight is attributable to cavitation influence, whereby ultrasound disrupts long protein chains into smaller fragments. These oxidative decomposition and carbonization reactions lead to scission of the polymer backbone, thereby altering the mass of the solid residue [35]. Our findings are consistent with those of Malik et al. [41], where ultrasonication accelerates the thermal degradation of sunflower seed protein.

### 3.4. Effect of Ultrasonic Treatment on the Free Sulfhydryl Groups and Disulfide Bonds of POPG

**Figure 4:**
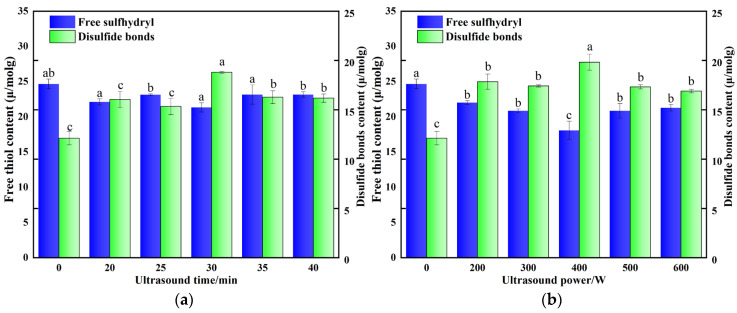
Effects of different ultrasonic treatments on free sulfhydryl and disulfide bonds of POPG: (**a**) Effects of different ultrasonic treatments time on free sulfhydryl and disulfide bonds of POPG; (**b**) Effects of different ultrasonic treatments power on free sulfhydryl and disulfide bonds of POPG. Different letters in the same column indicate significant differences (*p* < 0.05).

Free sulfhydryl groups can react with other sulfhydryl groups or disulfide bonds to form new disulfide bonds, facilitating the linkage of protein molecules and thus enhancing the network structure and gel strength. Figure 4a,b illustrates that the levels of free sulfhydryl groups and disulfide bonds of POPG after ultrasonic treatment were significantly different from those of the control samples. The free sulfhydryl content was the lowest, decreasing from 24.67 μmol/g to 18.07 μmol/g, while the disulfide bond content was the highest, increasing from 12.12 μmol/g to 19.83 μmol/g, which was at 400 W ultrasonic for 30 min. This phenomenon is related to the fact that the cavitation effect and mechanical shear force induced through ultrasonic treatment expose the concealed free sulfhydryl groups and disulfide bonds in POPG, which are then oxidized to form disulfide bonds, thereby improving the gel strength [42,43]. The alterations in free sulfhydryl and disulfide bonds can effectively validate the changes in gel strength induced by ultrasound. Our findings are consistent with Xin et al. [44], who reported that ultrasound treatment altered the amount of free sulfhydryl groups and disulfide bonds of gels formed by salted egg white and cooked soy protein isolate. However, excessive ultrasonic treatment can break some of the disulfide bonds that have already formed, leading to a loose structure and weakening its network structure [45].

### 3.5. Effect of Ultrasonic Treatment on the Surface Hydrophobicity of POPG

**Figure 5:**
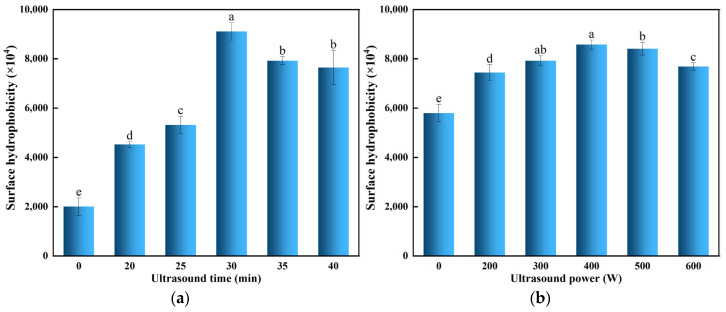
Effects of different ultrasonic treatments on surface hydrophobicity of POPG: (**a**) Effects of different ultrasonic treatments time on surface hydrophobicity of POPG; (**b**) Effects of different ultrasonic power treatments on surface hydrophobicity of POPG. Different letters in the same column indicate significant differences (*p* < 0.05).

Surface hydrophobicity is essential for maintaining the conformation and functional characteristics of proteins [12]. Figure 5a,b illustrate that, relative to the untreated POPG, the surface hydrophobicity of ultrasonicated POPG initially increases, followed by a decrease under varying ultrasonic conditions. This was due to the cavitation effect and shear forces produced by ultrasonic treatment that break the non-covalent bonds of POPG and facilitate the partial unfolding of proteins, while consequently leading to the hydrophobic groups buried inside polypeptide chains being exposed [46]. However, excessive ultrasonic treatment can cause the re-aggregation of POPG molecules and the hydrophobic groups to be reburied, thereby decreasing the surface hydrophobicity. Our findings are consistent with Chandrapala et al. [47], who reported that excessive ultrasound induces re-aggregation of whey proteins, reducing surface hydrophobicity.

### 3.6. Effect of Ultrasonic Treatment on the Intermolecular Forces of POPG

Intermolecular forces, specifically ionic bonds, hydrogen bonds, and hydrophobic interactions, are essential for gel formation [48]. As shown in Table 2, the ionic bonds and hydrogen bonds exhibited poor solubility stability, suggesting their secondary role in stabilizing the structural conformation of POPG. In contrast, the solubility stability of hydrophobic interactions greatly exceeds that of ionic and hydrogen bonds, which are the primary forces for the formation of POPG. The hydrophobic interactions of ultrasonicated POPG samples exhibited a preliminary increase succeeded by a reduction under varying ultrasonic conditions compared to the untreated, which are consistent with the change of the surface hydrophobicity. When the treatment time was 30 min, the hydrophobic interactions increased from 0.765 to 0.965, and when the power was 400 W, it reached 1.240. This can be attributed to the fact that moderate ultrasonic treatment can enhance hydrophobic interactions [49]. However, excessive ultrasonic treatment disrupts the molecular structure among POPG molecules and reduces the content of free thiol, consequently leading to a decline in hydrophobic interactions [50].

### 3.7. Effects of Ultrasonic Treatment on the Fluorescence Intensity of POPG

**Figure 6:**
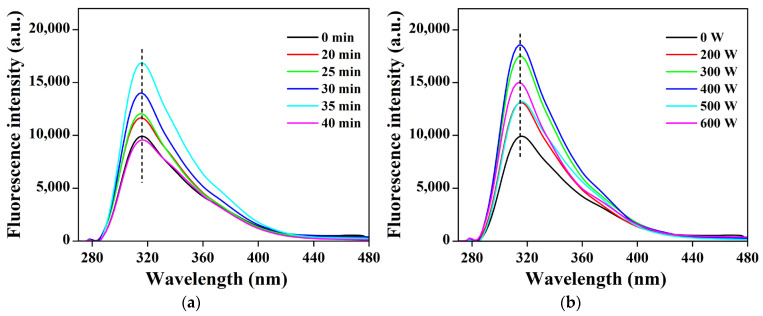
Effects of different ultrasonic treatments on fluorescence intensity of POPG: (**a**) Effects of different ultrasonic treatments time on fluorescence intensity of POPG; (**b**) Effects of different ultrasonic treatments power on fluorescence intensity of POPG.

Protein thermal unfolding can be quantified by the fluorescence intensity of residual sites. Tryptophan moieties are excited at 280 nanometers, so the fluctuations in their fluorescence intensity can reveal alterations in the tertiary structure of the protein [51]. As shown in Figure 6a,b, the fluorescence spectrum of ultrasonicated POPG aligned with those of untreated and exhibited no red or blue shifts. However, the intensity initially increased, followed by a decrease with increasing ultrasonic power and time. The highest fluorescence intensity of POPG was observed with ultrasonic 400 W for 35 min. This is due to moderate ultrasonic treatment facilitating the rearrangement of non-covalent bonds in protein molecules, exposing buried tryptophan chromophores and increasing fluorescence intensity [52]. However, excessive ultrasonic treatment causes protein molecules to aggregate and form particles, resulting in a significant decrease in the fluorescence signal [53].

### 3.8. Fourier Transform Infrared Spectra (FTIR) Analysis of POPG

**Figure 7:**
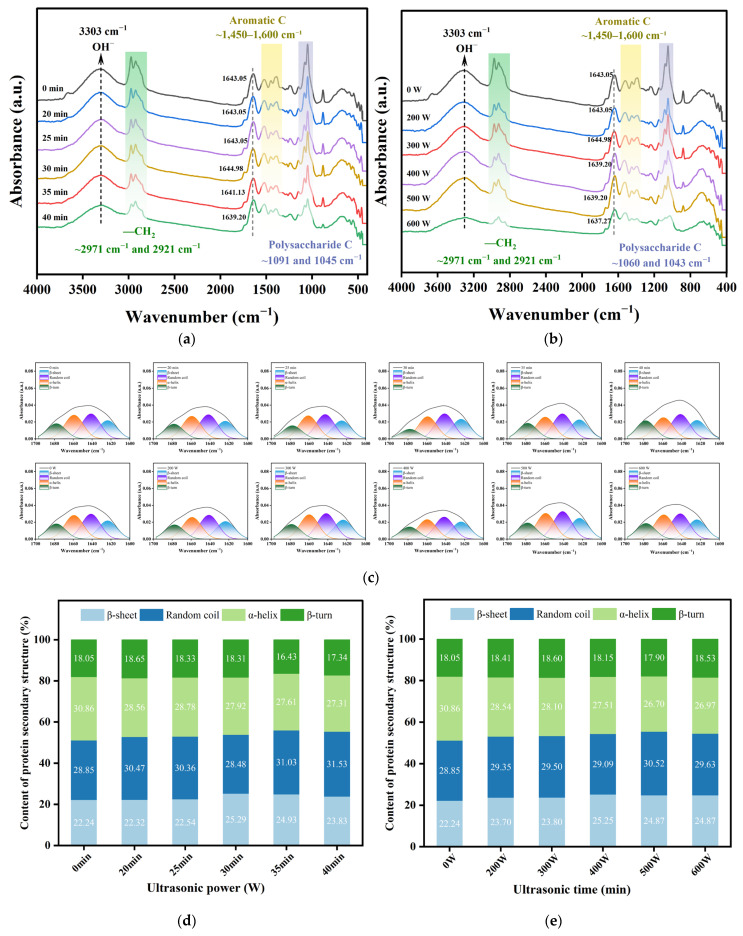
FTIR spectrum and distribution of protein secondary structure content: (**a**) FTIR spectrum of POPG with different ultrasonic treatments time; (**b**) FTIR spectrum of POPG with different ultrasonic treatments power; (**c**) Amide I band deconvolution of all the samples; (**d**) Distribution of protein secondary structure content of POPG with different ultrasonic treatments time; (**e**) Distribution of protein secondary structure content of POPG with different ultrasonic treatments power.

The alterations in the main conformation and secondary structure of the protein were assessed using infrared spectral analysis of POPG. As shown in Figure 7a,b, the broad peak at 3303 cm^−1^ is associated with the stretching vibration of O-H and N-H [54], while the typical characteristic peak of -CH_2_ is 2971 cm^−1^ and 2921 cm^−1^. The bending vibration of N-H and C=O generates the broad peak at 1600 cm^−1^ and 1450 cm^−1^ [55]. The inclusion of chitosan during the POPG preparation procedure is the cause of the more noticeable polysaccharide C at 1091 cm^−1^ and 1045 cm^−1^.

The spectrum reflecting the protein’s secondary structure was dispersed between 1600 cm^−1^ and 1700 cm^−1^ (amide I band). Figure 7a,b reveal that the amide I band of the ultrasonically treated sample exhibited varying degrees of subtle red shift, suggesting a potential alteration in the protein’s secondary structure. Gursel et al. [56] conducted analogous treatment on bovine serum albumin and observed a shift in the peak position of the amide-I region following ultrasonic treatment. Figure 7c displays the spectrum of materials subjected to various ultrasonic treatments within the amide I band (1600 cm^−1^–1700 cm^−1^). Figure 7d,e illustrate that under ultrasonic conditions of 400 W, the α-helix content diminishes greatly from 30.85% (untreated) to 27.51%. The α-helix mostly consists of intramolecular hydrogen bonds, which minimally contribute to the three-dimensional network, while ultrasonic wave cavitation results in the disruption of hydrogen bonds [57]. Conversely, the β-sheet content peaked, and the ultrasonic wave facilitated the conversion of α-helix to β-sheet structure, likely due to the ultrasonic wave augmenting protein interactions and altering protein structure [58]. Comparable phenomena were also noted when the ultrasonic treatment duration was 35 min. The augmentation of β-sheet content is pivotal for gel aggregation and network development, and the discovery of the microstructure confirms this.

### 3.9. Effect of Ultrasonic Treatment on the Microstructure of POPG

**Figure 8:**
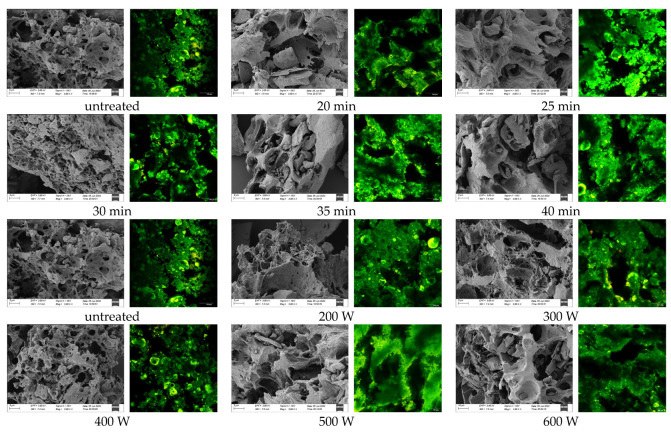
Effects of different ultrasonic treatments on SEM images of POPG: the left is the SEM image and the right is the CLSM image of each sample.

As shown in Figure 8, each pair of panels comprises an SEM image (left) and a CLSM image (right), the SEM image of ultrasonicated POPG samples, compared with the untreated samples, exhibited a more homogeneous and tight microstructure under varying ultrasonic conditions. Ultrasonic treatment causes smaller protein aggregates through mechanical forces, increasing the specific surface area [59]. The gel network structure is denser and more uniform with ultrasonic at 400 W for 30 min, exhibiting a reduced porosity microstructure. This improvement resulted in the exposure of functional groups in POPG, which promoted the formation of soluble protein polymers that filled network structure pores and improved gel network density [49,60]. However, excessive treatment may result in an enlarged size and uneven distribution of the gel structure due to excessive protein aggregation or denaturation [53], which covered active groups and reduced the surface area of POPG.

According to the analysis of the CLSM image (the right one in each pair of images in Figure 8), the green region represents the protein area, whereas the black region denotes the pores created between the protein structures following ultrasonic therapy. We observe that an increase in ultrasonic duration and strength results in smaller, more uniform, and denser holes within the protein structures. The image indicates that the sample treated for 30 min at 400 W exhibited optimal performance; however, with prolonged treatment time and increased power, larger black holes emerged, likely resulting from excessive ultrasound leading to protein molecule aggregation and disruption of the network structure [61]. A comparable phenomenon was likewise detected in the scanning electron microscope images.

The SEM and CLSM images indicate that with an ultrasonic power of 400 W and a treatment duration of 30 min, a dense and compact three-dimensional network structure was detected. This microscopic characteristic aligned with the assessment of gel strength, viscoelasticity, and thermal stability conducted before, and further corroborated the trend of intermolecular forces and protein secondary structure alterations under these conditions. Furthermore, the densely cross-linked protein framework depicted in the figure shows a substantial reduction in free sulfhydryl groups, a boost in disulphide bond content, and a slight increase in surface hydrophobicity. Nearly all of them support our hypothesis that “ultrasound can enhance the properties of POPG”. These outcome findings are consistent with Zhao et al., who reported that ultrasonic treatment leads to a denser and more uniform microstructure of soy protein gel [62].

## 4. Conclusions

This research investigated the effects of ultrasonic treatment on the characteristics of POPG by adjusting ultrasonic power (200–600 W, 30 min) and ultrasonic time (20–40 min, 400 W). Results showed that, with increasing ultrasonic time and power, the gel strength, WHC, free sulfhydryl content, surface hydrophobicity, and endogenous fluorescence intensity of POPG all exhibited a first increasing and then decreasing trend. When the ultrasonic parameters were set to 400 W and 30 min, all the aforementioned performance indicators of POPG reached their maximum values and were markedly superior to the control samples. Rheological analysis indicated that POPG displayed the highest storage modulus and better viscoelasticity. The thermal stability was significantly improved as confirmed by DSC and TGA. Analysis of intermolecular forces revealed that ultrasonic treatment facilitated the ordered aggregation of protein molecules by enhancing the hydrophobic interactions in POPG. The infrared spectrum indicates that ultrasonic therapy can marginally red-shift the peak of the protein amide I band, revealing the protein’s secondary structure. The augmentation of β-sheet is a significant factor contributing to the improvement of gel structure. This was further validated by observations using SEM and CLSM, which revealed a homogeneous and dense microstructure in POPG. This study indicated that the ultrasonicated POPG, considered a potential ingredient, improves functionality for many food applications in the food sector, contributing to the development of sustainable and nutritious plant-based products.

## Figures and Tables

**Table 1:** Effect of ultrasonic treatments on the thermal characteristics of POPG.

Ultrasonic Conditions		T_o_/°C	T_p_/°C	T_d_/°C	ΔH/J·g^−1^
Time (min)	Control	62.37 ± 1.95 ^d^	89.76 ± 1.13 ^c^	101.88 ± 1.57 ^c^	1695.75 ± 7.55 ^c^
	20	70.66 ± 1.79 ^c^	97.36 ± 1.11 ^b^	108.11 ± 1.24 ^bc^	1724.45 ± 18.85 ^bc^
	25	82.64 ± 2.22 ^b^	101.16 ± 1.42 ^ab^	108.82 ± 1.12 ^b^	1775.65 ± 19.75 ^ab^
	30	91.51 ± 0.97 ^a^	106.21 ± 2.03 ^a^	117.85 ± 4.14 ^a^	1822.35 ± 31.85 ^a^
	35	83.20 ± 1.77 ^b^	101.72 ± 1.18 ^ab^	110.44 ± 1.09 ^b^	1776.30 ± 8.90 ^ab^
	40	79.01 ± 1.56 ^b^	97.24 ± 1.91 ^b^	105.78 ± 1.14 ^bc^	1774.15 ± 28.45 ^ab^
Power (W)	Control	62.38 ± 1.95 ^c^	89.77 ± 1.13 ^c^	101.89 ± 1.57 ^c^	1695.75 ± 7.55 ^b^
	200	73.21 ± 2.89 ^b^	97.16 ± 1.74 ^b^	107.41 ± 1.98 ^bc^	1701.30 ± 19.90 ^b^
	300	77.30 ± 3.10 ^b^	98.84 ± 1.99 ^b^	108.56 ± 2.09 ^b^	1772.15 ± 18.25 ^ab^
	400	92.69 ± 2.63 ^a^	110.99 ± 2.33 ^a^	130.73 ± 2.04 ^a^	1842.35 ± 51.05 ^a^
	500	75.56 ± 3.89 ^b^	93.04 ± 2.72 ^bc^	105.75 ± 0.55 ^bc^	1835.15 ± 48.75 ^a^
	600	57.77 ± 2.55 ^c^	88.00 ± 1.68 ^c^	109.35 ± 2.03 ^b^	1726.65 ± 18.55 ^ab^

Different letters in the same column indicate significant differences (*p* < 0.05).

**Table 2:** Effect of ultrasonic on the intermolecular forces of POPG.

Ultrasonic Conditions		Ionic Bonds	Hydrogen Bonds	Hydrophobic Interactions
Time (min)	Control	0.017 ± 0.010 ^ab^	0.131 ± 0.004 ^b^	0.765 ± 0.016 ^c^
	20	0.024 ± 0.001 ^b^	0.105 ± 0.001 ^b^	0.739 ± 0.008 ^c^
	25	0.023 ± 0.001 ^ab^	0.149 ± 0.006 ^b^	0.791 ± 0.022 ^bc^
	30	0.020 ± 0.005 ^ab^	0.199 ± 0.010 ^a^	0.965 ± 0.020 ^a^
	35	0.024 ± 0.006 ^ab^	0.127 ± 0.001 ^b^	0.740 ± 0.036 ^c^
	40	0.048 ± 0.038 ^a^	0.219 ± 0.010 ^a^	0.848 ± 0.048 ^b^
Power (W)	Control	0.017 ± 0.010 ^c^	0.131 ± 0.004 ^d^	0.765 ± 0.016 ^d^
	200	0.016 ± 0.006 ^c^	0.282 ± 0.009 ^a^	1.026 ± 0.014 ^bc^
	300	0.068 ± 0.009 ^a^	0.206 ± 0.012 ^bc^	1.031 ± 0.029 ^bc^
	400	0.036 ± 0.004 ^b^	0.179 ± 0.011 ^cd^	1.240 ± 0.078 ^a^
	500	0.068 ± 0.133 ^a^	0.244 ± 0.008 ^ab^	0.995 ± 0.048 ^c^
	600	0.017 ± 0.010 ^c^	0.131 ± 0.004 ^d^	0.765 ± 0.016 ^d^

Different letters in the same column indicate significant differences (*p* < 0.05).

## Data Availability

The original contributions presented in the study are included in the article. Further inquiries can be directed to the corresponding authors.

## Abbreviations

The following abbreviations are used in this manuscript:

POPG	Pea–oat Protein Gel
TG	Transglutaminase
DSC	Differential Scanning Calorimetry
TGA	Thermogravimetric Analysis
FTIR	Fourier Transform Infrared Spectra
SEM	Scanning Electron Microscope
CLSM	Confocal Laser Scanning Microscope
